# CATH v4.4: major expansion of CATH by experimental and predicted structural data

**DOI:** 10.1093/nar/gkae1087

**Published:** 2024-11-20

**Authors:** Vaishali P Waman, Nicola Bordin, Andy Lau, Shaun Kandathil, Jude Wells, David Miller, Sameer Velankar, David T Jones, Ian Sillitoe, Christine Orengo

**Affiliations:** Institute of Structural and Molecular Biology, University College London, London WC1E 6BT, UK; Institute of Structural and Molecular Biology, University College London, London WC1E 6BT, UK; Department of Computer Science, University College London, London WC1E 6BT, UK; InstaDeep Ltd, 5 Merchant Square, London W2 1AY, UK; Department of Computer Science, University College London, London WC1E 6BT, UK; Institute of Structural and Molecular Biology, University College London, London WC1E 6BT, UK; Centre for Artificial Intelligence, University College London, London WC1V 6BH, UK; Institute of Structural and Molecular Biology, University College London, London WC1E 6BT, UK; Centre for Artificial Intelligence, University College London, London WC1V 6BH, UK; Protein Data Bank in Europe, European Molecular Biology Laboratory, European Bioinformatics Institute, Hinxton, Cambridge, CB10 1SD, UK; Institute of Structural and Molecular Biology, University College London, London WC1E 6BT, UK; Department of Computer Science, University College London, London WC1E 6BT, UK; Institute of Structural and Molecular Biology, University College London, London WC1E 6BT, UK; Institute of Structural and Molecular Biology, University College London, London WC1E 6BT, UK

## Abstract

CATH (https://www.cathdb.info) is a structural classification database that assigns domains to the structures in the Protein Data Bank (PDB) and AlphaFold Protein Structure Database (AFDB) and adds layers of biological information, including homology and functional annotation. This article covers developments in the CATH classification since 2021. We report the significant expansion of structural information (180-fold) for CATH superfamilies through classification of PDB domains and predicted domain structures from the Encyclopedia of Domains (TED) resource. TED provides information on predicted domains in AFDB. CATH v4.4 represents an expansion of ∼64 844 experimentally determined domain structures from PDB. We also present a mapping of ∼90 million predicted domains from TED to CATH superfamilies. New PDB and TED data increases the number of superfamilies from 5841 to 6573, folds from 1349 to 2078 and architectures from 41 to 77. TED data comprises predicted structures, so these new folds and architectures remain hypothetical until experimentally confirmed. CATH also classifies domains into functional families (FunFams) within a superfamily. We have updated sequences in FunFams by scanning FunFam-HMMs against UniProt release 2024_02, giving a 276% increase in FunFams coverage. The mapping of TED structural domains has resulted in a 4-fold increase in FunFams with structural information.

## Graphical Abstract

**Graphical Abstract:**
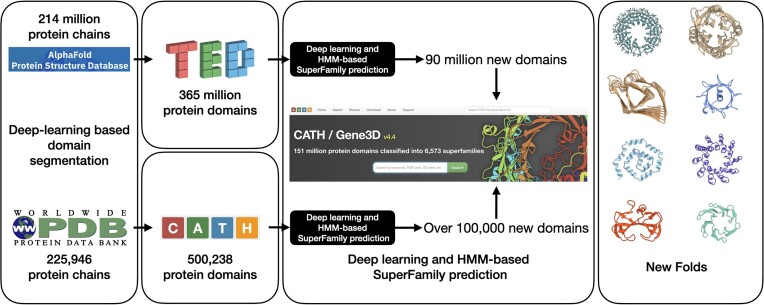

## Introduction

CATH (https://www.cathdb.info) is a structural classification database developed in 1997 (1), that assigns domains to the structures available in the PDB (2) and the AlphaFold Protein Structure Database (AFDB) (3) and adds layers of biological information, including homology and functional annotation. Domains in CATH are classified into the following hierarchical levels: Class (C), Architecture (A), Topology (T) and Homologous superfamilies (H) (1,4,5). CATH is a Core Data Resource within ELIXIR, a major European distributed infrastructure for life-science information (https://elixir-europe.org/platforms/data/core-data-resources), and has recently been endorsed as a Global Core BioData Resource (GCBR) by the Global Biodata Consortium (https://globalbiodata.org/).

Protein structures are segmented into their constituent domains (semi-independently folding globular units) for classification in CATH, using semi-automated approaches (6)]. Since AFDB (https://alphafold.ebi.ac.uk/) has ∼1000-fold more entries than PDB our workflow for automated segmentation of protein domains has been expanded to include a much faster and more accurate in-house deep-learning approach (Chainsaw (7)) and two other publicly available methods (Merizo (8), developed by the Jones group, and UniDoc (9)).

We have also developed a new deep-learning based tool for homologue detection; CATHe (10). Furthermore, our suite of protein structure comparison tools and associated workflow, used for homologue detection and verification (SSAP (11), CATHEDRAL (6)), has been expanded to include the publicly available state-of-the-art tools namely Foldseek (12) and Foldseek-TMalign (12), developed by the Steinegger group and Merizo-search (13), developed by the Jones group.

Domain sequences in UniProt (14) predicted to belong to CATH superfamilies are available from our sister resource, Gene3D (15)). We use sequences from representative structural domains from each CATH superfamily to generate multiple sequence alignments, which are converted into hidden Markov models (HMMs) (16). These HMMs are then used to identify closely related domains within protein sequences from UniProt (14) and ENSEMBL (17). More recently, a new resource (TED (18)) established by automated protocols developed by the groups of Jones and Orengo, provides information on domains in protein structures predicted by AlphaFold2 (19). Gene3D and TED are sister resources to CATH, comprising predicted CATH family annotations. As such they extend knowledge on likely sequence and structure diversity in CATH superfamilies. However, since they are predicted domains and annotations, whilst the data are linked to CATH superfamilies, they are not formally integrated in the CATH web pages until experimental verification is obtained.

Domain structures are classified in CATH superfamilies provided we have evidence from two or more independent approaches, e.g. a structure-based match from Foldseek to one or more relatives in the superfamily and a sequence-based match from HMMER3 or CATHe. In CATH 4.4 we provide a mapping of a significant subset (27.8%) of high-quality TED domains to CATH superfamilies (based on Foldseek and HMM-based matches where both methods agree on the superfamily prediction and the boundary overlap is 80%).

CATH also subclassifies superfamilies into functional families (FunFams) using a hierarchical agglomerative clustering algorithm (20) which segregates functional families on the basis of differentially conserved specificity-determining positions (SDPs) (21). For each FunFam, CATH provides multiple sequence alignments, HMMs and high-quality GO annotations from UniProt-GOA (22).

We report the significant expansion of structural information associated with CATH from experimental domain structures (from the PDB) and predicted domain structures (from AFDB). This data increases our knowledge of evolutionary superfamilies, fold groups and architectures in protein space, although it is important to note that the data mapped from TED are predicted and need to be confirmed experimentally. We also report the expansion of domain entries in FunFams and the increase in structural coverage of FunFams by predicted TED structures.

## CATH 4.4 release highlights

### Expansion of CATH superfamilies with newly classified domain structures from the PDB

As reported in Waman *et al.* (25) we recently applied our in-house Chainsaw algorithm (7) to segment protein structures in the PDB not yet classified in CATH. These domains were subsequently scanned against non-redundant representatives (95% sequence identity, S95 reps) from CATH using Foldseek to identify putative superfamily or fold matches. Homology was verified by scanning against the HMM libraries for the matched superfamily and by application of CATHe (10). This process allowed us to bring 64 844 domain structures from PDB structures into 1361 existing CATH superfamilies and to identify 250 new folds to CATH. Class and architecture annotations have been manually assigned to these new folds (see Supplementary File 1), identifying two additional architectures, see section 2.0 below). The newly classified domains are now integrated in CATH and can be viewed on the CATH 4.4 web pages.

### Expansion of structural data in CATH superfamilies with predicted domain structures from AFDB

Since the release of CATH 4.3, major developments in protein structure prediction (AlphaFold2 (19)) have led to the establishment of the AlphaFold Protein Structure Database (AFDB), comprising 214 million protein structures for the vast majority of UniProt entries (version 2021_04 (14)). Evaluation by CASP14 (23) established AlphaFold2 as a leading structure prediction method and endorsed the quality metrics reported by the algorithm.

The Jones and Orengo groups at UCL have collaborated over the last year to process the predicted protein structures in AFDB. An automated consensus protocol was developed to segment these structures into globular domains. Segmented domains were subsequently mapped to CATH superfamilies and fold groups. Those domains with no similarity to CATH superfamily or fold group relatives were identified as potential novel folds. Information on the AFDB TED domains, including domain boundaries, model quality, annotated CATH superfamily/fold group or assignment of novel fold groups are presented in the TED (**T**he **E**ncyclopedia of **D**omains) resource (18).

Below we briefly summarise the TED protocol and report on the number of domains that have been assigned to CATH superfamilies using both structure and sequence matching.

#### Domain segmentation and classification of TED domains into CATH superfamilies and fold groups

Domain segmentation of AFDB structures for TED was based on a consensus protocol (18) (which seeks agreement from at least 2 out of 3 domain segmentation methodsnamely Chainsaw (7), Merizo (8), Unidoc (9)). The TED protocol subsequently clusters all the domains detected by the consensus segmentation into sequence clusters (at 50% sequence identity) using MMseqs (24). Cluster representatives are subjected to various quality filters (see (18) for further details) and scanning against CATH S95 superfamily representatives by Foldseek (12) using established thresholds for homology and fold similarity (25). Only domains with good quality models (pLDDT ≥ 70) are considered for mapping to CATH.

**Figure 1.**
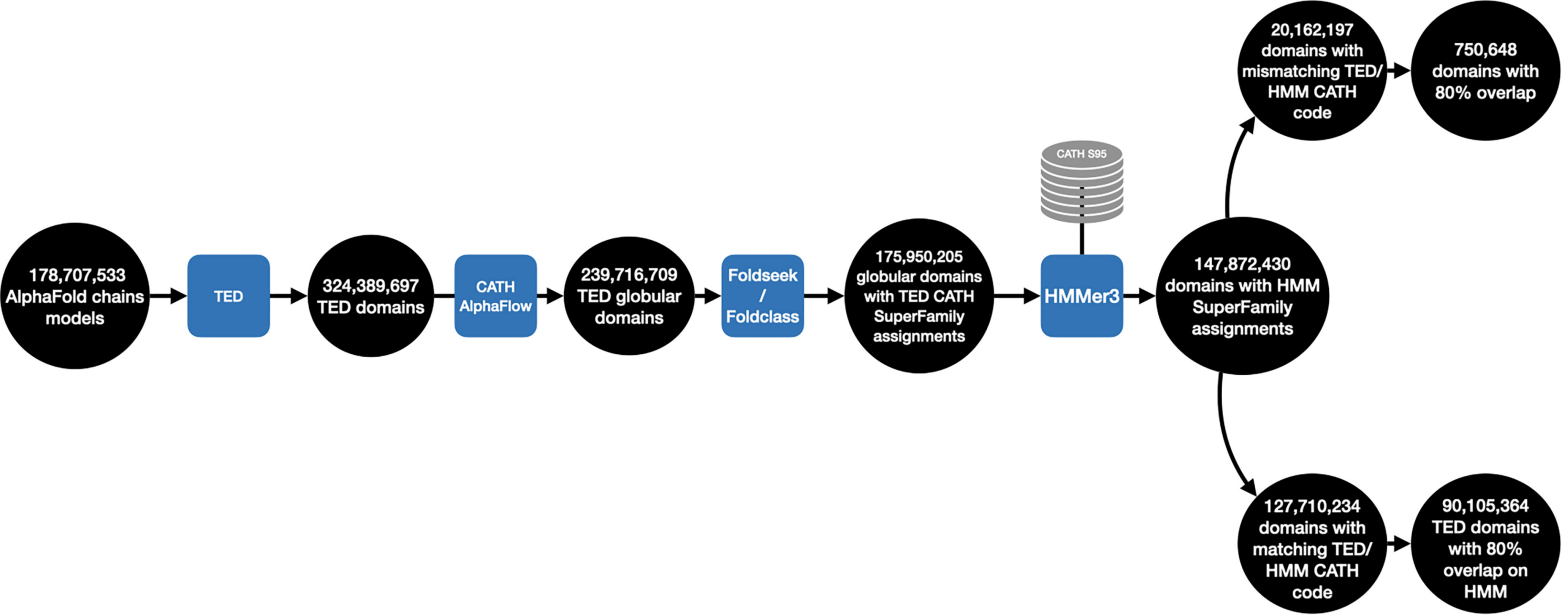
Overview of domain counts at each step of the CATH homology assignment protocol for TED domains. TED domains with predicted globularity, with at least six secondary structure elements predicted by STRIDE and pLDDT over 70 are scanned with HMMER3 against a library of Hidden Markov Models (HMMs) built from CATH representatives (from clusters of sequences at 95% sequence identity), with boundaries overlap of 80% and above being considered for confident CATH assignments.

Our established CATH superfamily classification protocol assigns domains to a superfamily provided they have significant structural similarity and a significant HMM match to one or more relatives in that superfamily. Therefore, for TED domains mapped to a CATH superfamily by Foldseek we used HMMER3 to scan the TED domains against the HMM library for all CATH superfamilies (generated for Gene3D version 22). 84% of TED domains annotated with CATH superfamilies by Foldseek have a significant HMM match but 13% of these did not match the superfamily identified by Foldseek but matched another superfamily (from a similar fold group (T) or architecture (A)) (see Figure 1). This may indicate an evolutionary relationship between the two superfamilies which will require manual verification. However, <10% of these TED/HMM matches agree in domain boundaries so this set may also contain domains misassigned by one or other method.

**Figure 2.**
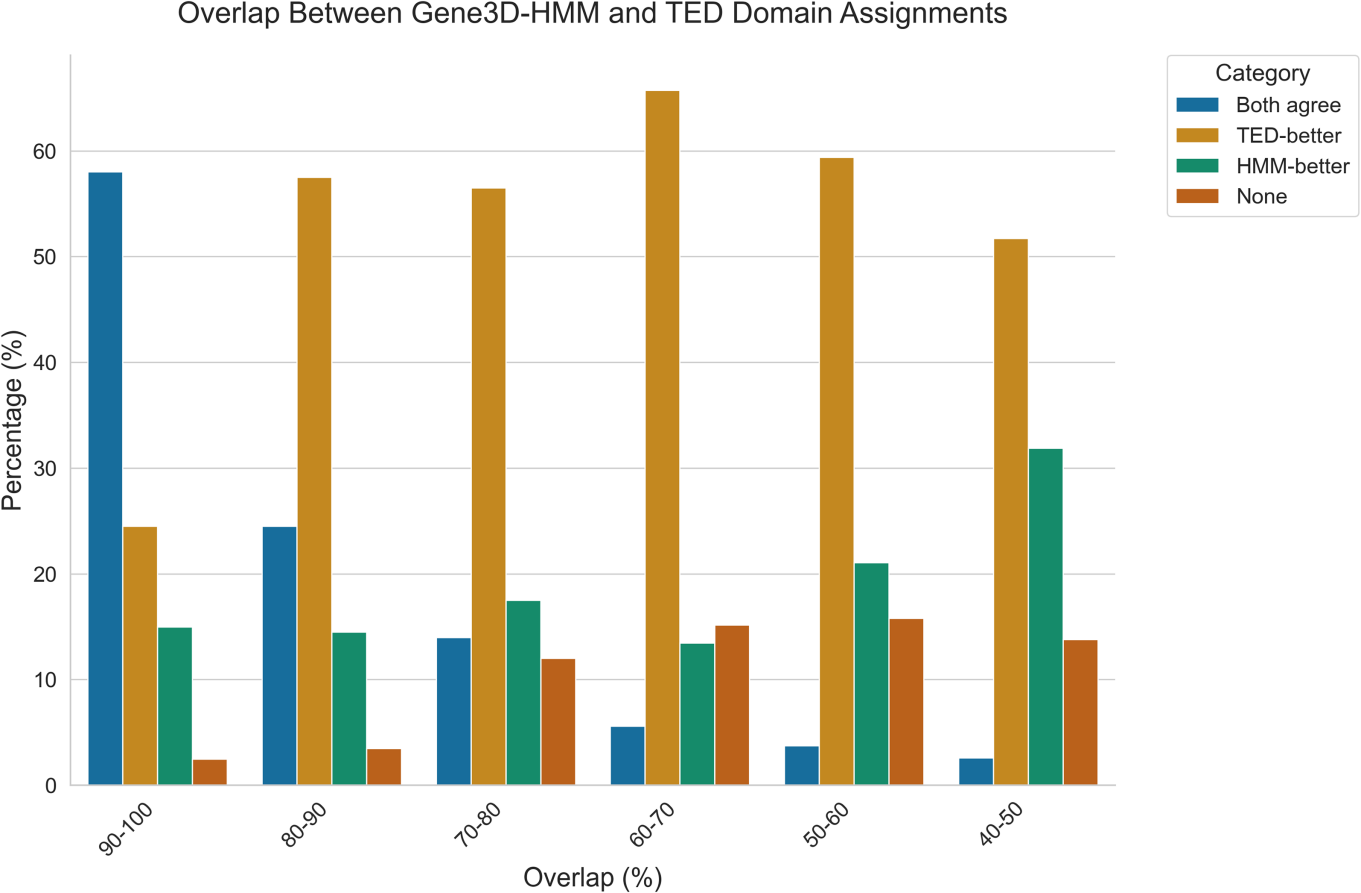
Performance of Gene3D(HMM) and TED protocols in assigning domain boundaries.

Manual curation of 1200 TED domains mapped to CATH superfamilies found that for domains with good overlap (≥80%) between the boundaries assigned by TED and HMM, domains were well defined by both algorithms. TED boundaries were typically better (see Figure 2). At this level of overlap, discrepancies between HMM and TED boundaries typically involve one or two residues at the termini of the domain with an overall error rate <5%.

We identified 90 105 364 TED domains with highly overlapping HMM assignments (≥80% overlap), and where superfamily assignments by TED/HMM agree. Information on these domains can be downloaded from the CATH ftp site (ftp://orengoftp.biochem.ucl.ac.uk/cath/releases/latest-release/). The latest statistics for PDB (see Table 1) and TED (Table 2) domain structures assigned in CATH superfamilies in CATH v4.3 and v4.4, is provided below.

**Table 1.** CATH v4.4 statistics

Numbers/statistics	**CATH v4.3**	**CATH v4.4**
Domains from PDB	500 238	601 493
Superfamilies from PDB	5481	6631
Folds from PDB	1390	1472
Architectures from PDB	41	43
Number of domains in FunFams	34 700 216	96 078 753
FunFams with CATH structural domains from PDB	17 208	13 893

Summary of the number of experimental PDB domains, folds, architectures and FunFam domains in CATH v4.3 and v4.4.

**Table 2.** Summary of TED domain mappings to CATH superfamilies, hypothetical novel folds and architectures, identified from the TED data

**Numbers/statistics**	**CATH v4.3**	**CATH v4.4**
Number of domains from TED	-	90 105 364
Superfamilies from TED	-	479
Folds from TED	-	479
Architectures from TED	-	34
FunFams with TED domains	-	69 900

**Figure 3.**
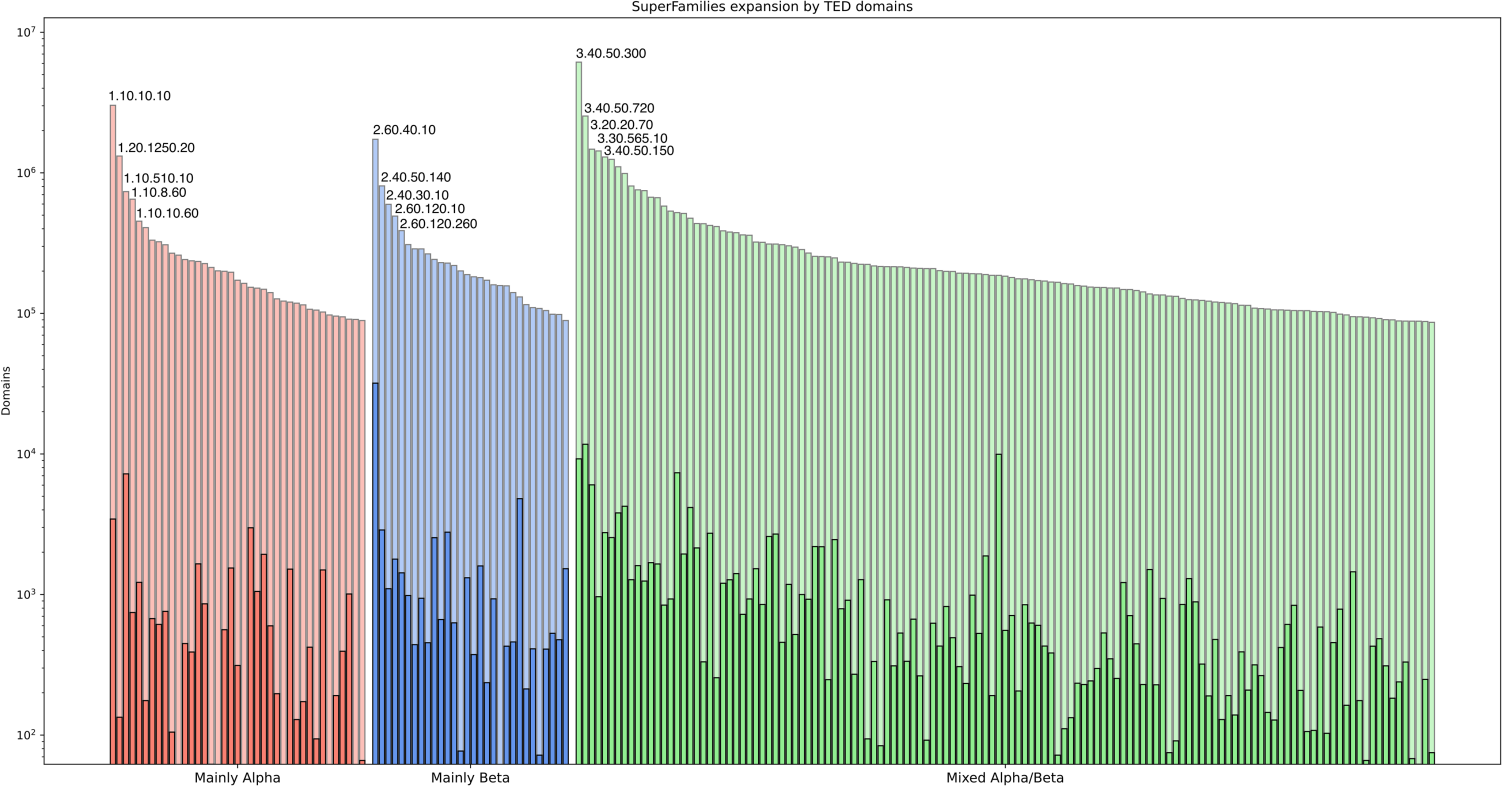
Expansion of CATH Superfamilies by structural information from TED domains. Top 200 Superfamilies expansion by TED domains (light) over existing PDB domains in CATH (dark) segregated by CATH classes, with the 5 most expanded Superfamilies per class indicated by their CATH classification ID.

Some CATH superfamilies are significantly expanded by the TED-HMM domains (Figure 3). In particular, membrane-associated structures which are less tractable for experimental determination. See (18) for a detailed discussion of all CATH superfamily annotations for TED predicted domain structures and preliminary structural and functional analyses of the data.

A further 85 million TED domains could be mapped to CATH superfamilies or fold groups by Foldseek and Merizo-search. Although there are no HMM matches to CATH superfamilies for these domains, future work will involve scanning these domains against HMMs built from the ∼90m TED-HMM domains assigned to CATH superfamilies based on TED/HMM assignments. We anticipate that further verification of many of these remote putative evolutionary relationships to CATH superfamilies will be obtained by scanning against these HMMs derived for the >200-fold larger dataset of CATH domains associated with version 4.4.

The TED resource (https://ted.cathdb.info) provides information on all TED domains mapped to CATH superfamilies using the TED Foldseek and Merizo-search based protocols (see (18) for more details). For each TED domain, information is provided on boundary predictions by all the segmentation methods (Chainsaw, Merizo, UniDoc) together with a visualisation of the whole protein structure allowing the user to compare between the predicted segmentations.

### Identification of novel superfamilies, fold groups and architectures in the TED data

A set of 13 860 TED domain cluster representatives did not match any CATH experimental superfamily/fold structures using Foldseek or Merizo-search, even using very liberal score thresholds, suggesting that these are hypothetical new folds.

**Figure 4.**
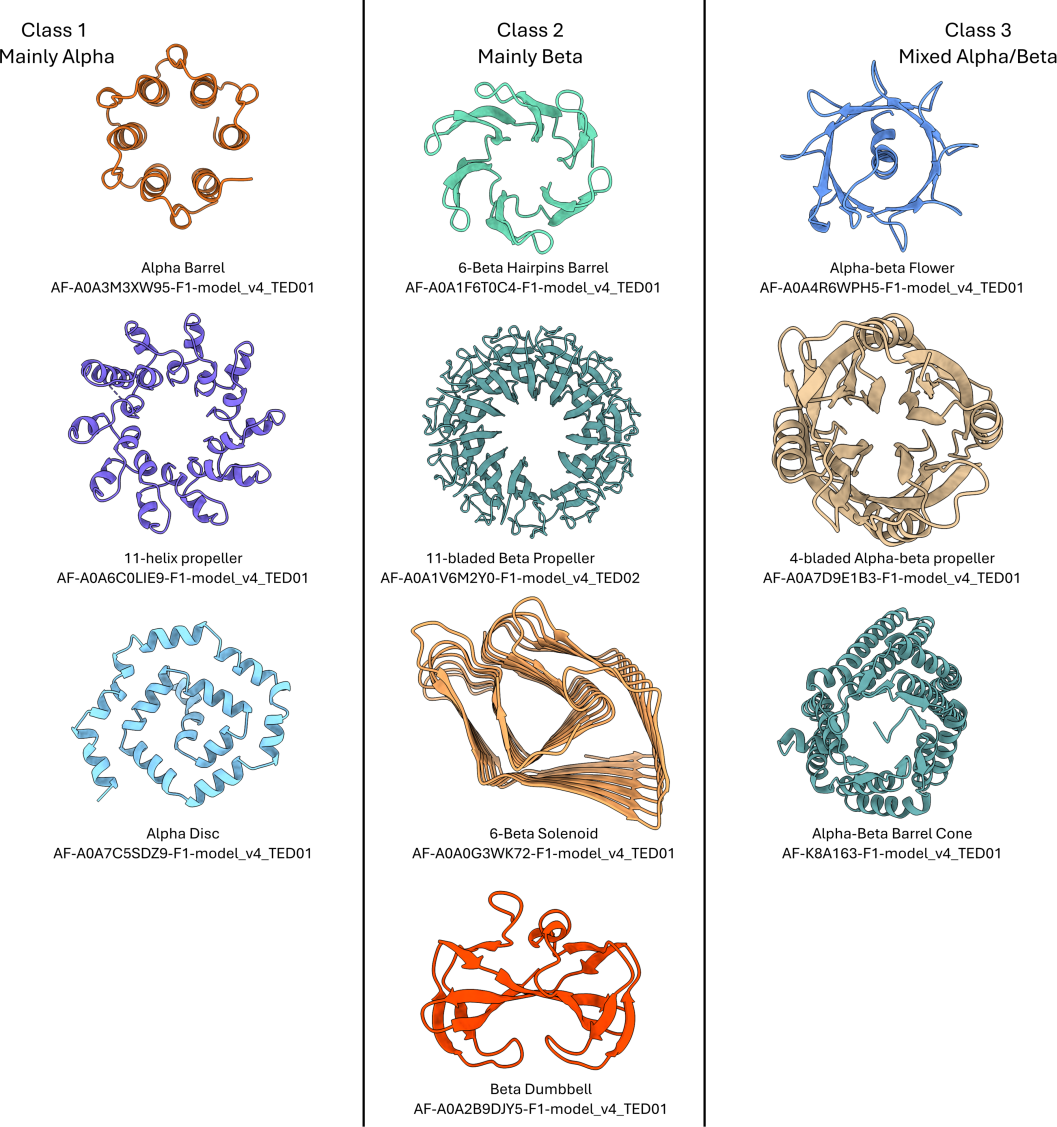
Illustration of a selected set of new architectures from TED, now classified in CATH v4.4.

New fold groups are manually curated in CATH to determine their class and architecture. To date 479 of these new folds have been assigned Class and Architecture categories (Supplementary File 1). During this process we identified and named 34 new architectures including in the Alpha Class (Alpha 11-helix propeller, Alpha Disc, (Single) Alpha Barrel), Beta class (Beta hairpins Barrel, 11-bladed beta propeller, 6-Beta Solenoid) and Alpha Beta class (e.g. Alpha-Beta flower, 4-bladed propeller, Alpha Beta-barrel cone), see selected representatives in Figure 4. It is important to note these new categories are hypothetical until experimentally confirmed.

### Expansion of domains in CATH functional families (FunFams) and increase in structural representatives

CATH superfamilies are sub-clustered into functional families (FunFams) using an agglomerative clustering approach that segregates clusters based on differentially conserved residues. There were 212 872 FunFams in CATH release 4.3. Sequences in each FunFam were aligned using MAFFT (26) and an HMM built from the multiple sequence alignment using HMMER3.

**Figure 5.**
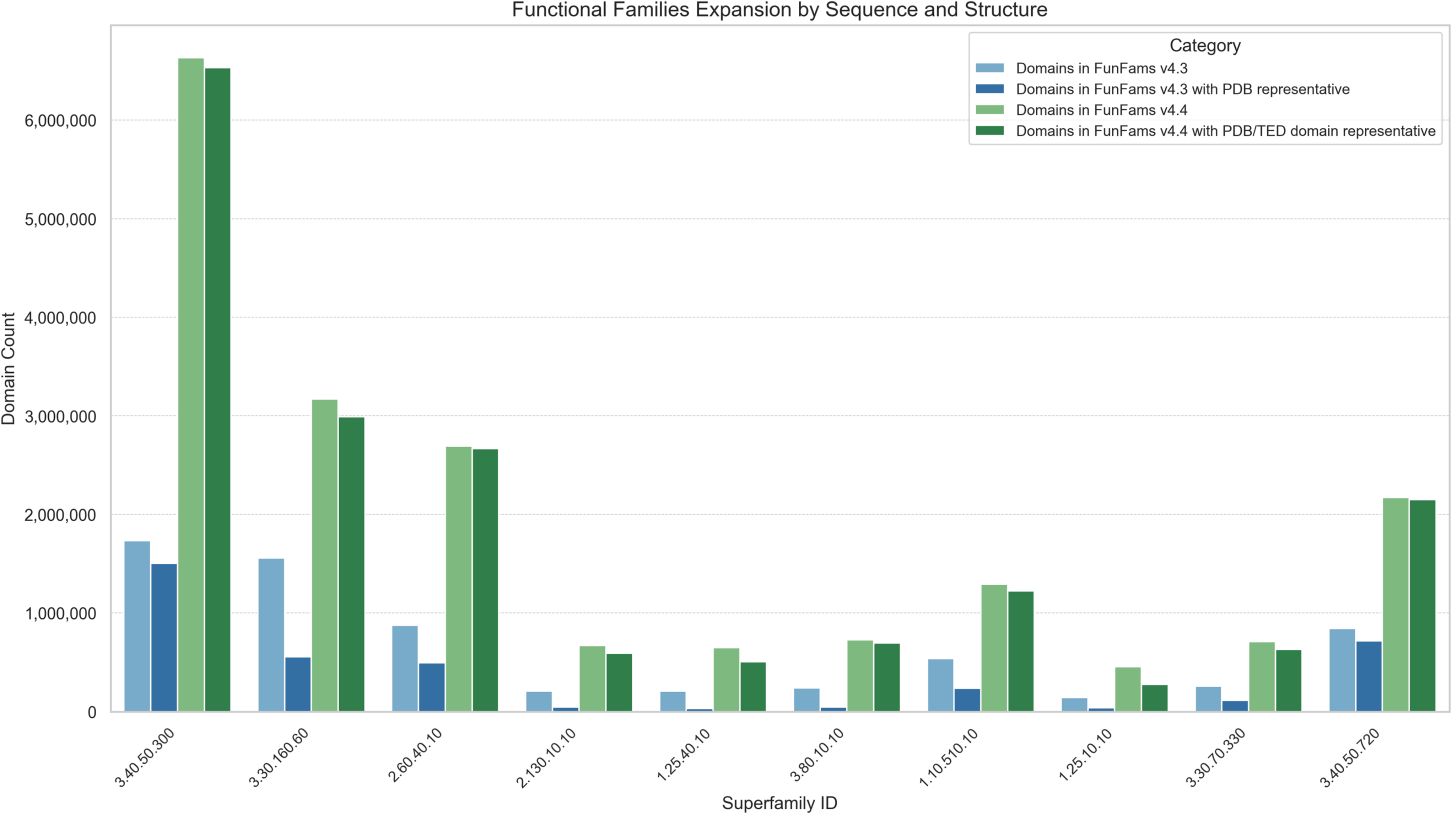
Expansion of 10 most highly populated CATH FunFams with UniProt domain sequences assigned by HMM. The increase in structural representation by TED predicted domain structures is also shown.

We have expanded the CATH FunFams by scanning domain sequences from UniProt release 2024_03 against the FunFam HMMs, increasing the total number of FunFam relatives from 34 700 216 in CATH v4.3 to 96 078 753 (276% increase). We also scanned the TED domain sequences against the FunFam HMMs resulting in a mapping of 44 767 099 TED domains to 69 990 CATH FunFams. This significant expansion of structural information in the CATH FunFams resulted in a more than 4-fold increase (from 17 208 to 73 215) in the number of FunFams having a structural representative. Figure 5 illustrates the top 10 most populated CATH FunFams expanded by TED.

There are now 73 215 FunFams with at least one good quality domain structure representative from the PDB or AFDB. We are currently building multiple sequence alignments of these to enable detection of highly conserved residues (27) which can be mapped to the structural relative to identify putative functional ‘hot’ spots.

## Conclusion

CATH has recently been recognised as a Global Core BioData Resource (GCBR) and is one of the few national resources to be endorsed in this way. As with our sister resource, Gene3D, which provides predicted domains in UniProt entries using HMM based assignments, the TED resource developed by the Jones and Orengo groups provides information on identified domains in the AFDB protein structures together with annotations for CATH superfamilies and novel fold groups. These annotations are based on automated algorithms associated with a certain error rate. The scale of the data is too vast for extensive manual curation. However, for ∼90 million domains we verify superfamily annotations using our established CATH HMM-based protocol (domains labelled as TED-HMM). Manual curation of a subset of 1200 domains confirms that TED domains are typically more accurate than HMM based assignments.

Our recent addition of domain structures from the PDB (101 255) and TED (90 105 364) resources has expanded the number of domain structures associated with CATH superfamilies by nearly 200-fold, to 90 124 482 domains and revealed 729 new fold groups (250 from PDB, 479 from TED) and 36 total new architectures (2 from PDB, 34 from TED) in CATH.

TED data will be continuously improved by evolving the algorithms and consensus workflows for segmenting the domains. Some of the common issues we detected in domain boundary assignments were problems in handling repeat structures, or AFDB structures in which relatively large portions of the domain structure had poor model quality. Furthermore, domains with large interfaces and tight packing with another domain in the same protein. Furthermore, domains with large interfaces and tight packing with another domain in the same protein were particularly challenging for our domain boundaries predictors. In some cases, segmented domains had been merged with close-packed structural fragments which are clearly not part of the domain fold but may have a role in promoting a domain or protein interaction.

We will continue to apply the established CATH classification protocols (i.e. in addition to structure based mapping by Foldseek and Merizo-search, HMM and sequence embedding based homologue detection, followed by manual curation for borderline matches and assignment of CATH class and architecture to new fold groups) to carefully map TED domains to CATH superfamilies and novel CATH fold groups. The expansion of information on CATH superfamilies and FunFams with high quality predicted domain structures will significantly improve analyses of structural mechanisms underpinning functional divergence across CATH superfamilies.

## Supplementary Material

gkae1087_Supplemental_File

## Data Availability

CATH website: https://www.cathdb.info, CATH FTP website: ftp://orengoftp.biochem.ucl.ac.uk/cath/releases/, TED resource: https://ted.cathdb.info.
